# Effect of Antimicrobial Exposure on AcrAB Expression in *Salmonella enterica* Subspecies *enterica* Serovar Choleraesuis

**DOI:** 10.3389/fmicb.2013.00053

**Published:** 2013-03-14

**Authors:** Masaru Usui, Hidetaka Nagai, Mototaka Hiki, Yutaka Tamura, Tetsuo Asai

**Affiliations:** ^1^National Veterinary Assay Laboratory, Ministry of Agriculture, Forestry and FisheriesKokubunji, Tokyo, Japan; ^2^School of Veterinary Medicine, Rakuno Gakuen UniversityEbetsu, Hokkaido, Japan

**Keywords:** AcrAB efflux pump, antimicrobial resistance, RamA, *Salmonella* Choleraesuis, SoxS

## Abstract

Understanding the impact of antimicrobial use on the emergence of resistant bacteria is imperative to prevent its emergence. For instance, activation of the AcrAB efflux pumps is responsible for the emergence of antimicrobial-resistant *Salmonella* strains. Here, we examined the expression levels of *acrB* and its multiple regulator genes (RamA, SoxS, MarA, and Rob) in 17 field isolates of *S*. Choleraesuis by using quantitative PCR methods. The expression of *acrB* increased in eight of the field isolates (*P* < 0.05). The expression of *acrB* was associated with that of *ramA* in one isolate, *soxS* in one isolate, and both these genes in six isolates. Thereafter, to examine the effect of selected antimicrobials (enrofloxacin, ampicillin, oxytetracycline, kanamycin, and spectinomycin) on the expression of *acrB* and its regulator genes, mutants derived from five isolates of *S*. Choleraesuis were selected by culture on antimicrobial-containing plates. The expression of *acrB* and *ramA* was higher in the mutants selected using enrofloxacin (3.3–6.3- and 24.5–37.7-fold, respectively), ampicillin (1.8–7.7- and 16.1–55.9-fold, respectively), oxytetracycline (1.7–3.3- and 3.2–31.1-fold, respectively), and kanamycin (1.6–2.2- and 5.6–26.4-fold, respectively), which are AcrAB substrates, than in each of the parental strains (*P* < 0.05). In contrast, in AcrAB substrate-selected mutants, the expression of *soxS*, *marA*, and *rob* remained similar to that in parental strains. Of the four antimicrobials, the level of *ramA* expression was significantly higher in the enrofloxacin- and ampicillin-selected mutants than in the oxytetracycline- and kanamycin-selected mutants (*P* < 0.05), whereas the expression levels of *acrB* and multiple regulator genes in spectinomycin-selected mutants were similar to those in each parental strain. These data suggest that exposure to antimicrobials that are AcrAB substrates enhance the activation of the AcrAB efflux pump *via* RamA, but not *via* SoxS, MarA, or Rob in *S*. Choleraesuis.

## Introduction

*Salmonella* *enterica* subspecies *enterica* serovar Choleraesuis is a bacterial pathogen that causes severe diarrhea, pneumonia, and septicemia in pigs and in elderly and immunocompromised humans (Chiu et al., 2004). These bacteria sometimes cause severe infections, necessitating antimicrobial treatment of affected patients. A high rate of multidrug resistance among *S*. Choleraesuis has been reported in several countries (Lee et al., 2009; Asai et al., 2010), raising a concern for public and animal health.

**Figure 1:**
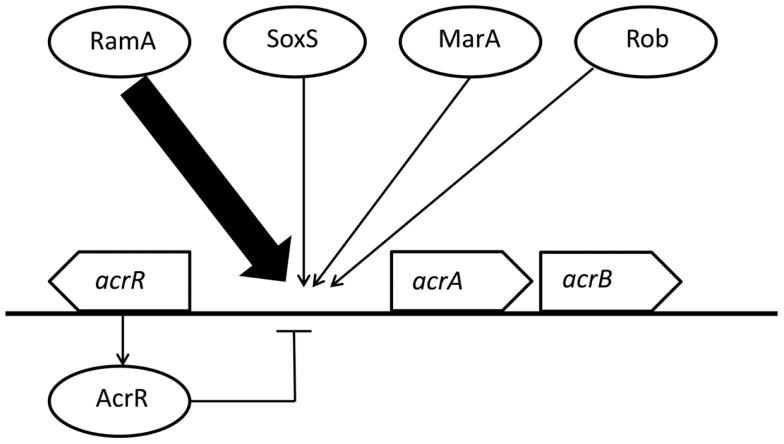
**Regulation of *acrAB* expression by multiple regulators in *Salmonella* Typhimurium and *S*. Haardt**. This figure was produced using data from the literature (see main text). Functional interactions are represented as arrows for activation/induction and as “⊣” for repression. The size of arrows indicates the estimated impact.

Activation of an efflux system that removes the drug from the cell is one antimicrobial resistance mechanism in bacteria (Chen et al., 2007; Li and Nikaido, 2009). The AcrAB-TolC system is the main multidrug efflux system in Gram-negative bacteria (Giraud et al., 2000). The expression level of *acrAB* mRNAs correlates with efflux activities and is regulated by global regulatory proteins and local repressors (Rosenberg et al., 2003; Li and Nikaido, 2004; Olliver et al., 2004; Abouzeed et al., 2008). In particular, the expression of *acrAB* is regulated by the global regulators, RamA, SoxS, MarA, and Rob (Rosenberg et al., 2003; Abouzeed et al., 2008). Moreover, mutations within the local repressor AcrR contribute to the overexpression of *acrAB* (Olliver et al., 2004). In *S*. Typhimurium and *S*. Haardt, RamA mainly regulates the expression of *acrAB* (Figure 1; Nikaido et al., 2008; Zheng et al., 2009; Kim and Woo, 2011). Other regulators, SoxS and MarA, are also known to regulate *acrAB* expression, but the contributions of SoxS and MarA to antimicrobial susceptibilities are lower than that of RamA (Abouzeed et al., 2008). Little is known about Rob and its contribution to the enhancement of AcrAB in *Salmonella* (Rosenberg et al., 2003). In *S*. Pullorum, the expression of *acrB*, which shifts from sensitivity to resistance against bile salt (deoxycholate, an AcrAB substrate), was independent of *ramA*, *soxS*, *marA*, and *rob* expression (Usui et al., 2011).

We have previously recorded the various efflux activity levels of *S*. Choleraesuis under field conditions, suggesting that elevated efflux activities are related to the emergence of fluoroquinolone resistance (Usui et al., 2009). In fluoroquinolone-selected *S*. Choleraesuis, efflux pumps including AcrAB were enhanced and resulted in decreasing susceptibilities to several antimicrobials (Usui et al., 2010). However, little is known about roles of regulator genes for efflux pumps in *S*. Choleraesuis. In this study, we examined the expression levels of *acrAB* and multiple regulator genes in 17 clinical swine isolates of *S*. Choleraesuis and antimicrobial-selected mutants in order to understand the impact of antimicrobial exposure on the efflux activities in this organism.

## Materials and Methods

### Bacterial strains

Seventeen strains of *S*. Choleraesuis, obtained from different diseased pigs between 2001 and 2005 (Asai et al., 2010), were used in this study (Table 1). These strains comprised 6 nalidixic acid-resistant strains (low enrofloxacin-accumulation), and 11 nalidixic acid-susceptible strains (two strains: low enrofloxacin-accumulation; three strains: intermediate enrofloxacin-accumulation; six strains: high enrofloxacin-accumulation) as previously recorded (Usui et al., 2009). The intracellular concentration of enrofloxacin was significantly lower in nalidixic acid-resistant isolates, and the nalidixic acid-susceptible isolates ZSC-8 and 582 as compared to other susceptible isolates with the exception of isolates 143, ZSC-12, and 1002 (*P* < 0.1; Usui et al., 2009). The intracellular enrofloxacin concentrations of isolates 143, ZSC-12, and 1002 were at an intermediate level. Minimum inhibitory concentrations (MIC) of enrofloxacin, ampicillin, oxytetracycline, and kanamycin had been determined in our previous study (Asai et al., 2010). MIC of spectinomycin was determined by the broth microdilution method according to CLSI guidelines (Clinical and Laboratory Standard Institute, 2008).

**Table 1:** **Characterization of *Salmonella* Choleraesuis used in this study**.

Strain no.	Susceptibility to nalidixic acid^a^		Enrofloxacin-accumulation		mRNA expression		Mutations	
	Category^b^	MIC (mg/L)^b^	Category^c^	(ng/10^5^cfu)^c^	*acrA*^d^	*acrB*^d^	AcrR^e^	Binding site
13-PLS-6	Resistance	256	Low	8.2	1.6 ± 0.2*	1.8 ± 0.2*	WT	WT
14-PLS-21	Resistance	>512	Low	8.7	3.9 ± 0.3*	2.7 ± 0.2*	WT	WT
16-PLS-45	Resistance	>512	Low	6.5	1.5 ± 0.1*	1.9 ± 0.3*	Q78ter	WT
16-PLS-46	Resistance	>512	Low	6.6	2.4 ± 0.2*	2.4 ± 0.4*	Q78ter	WT
sal-1372	Resistance	>512	Low	6.3	1.8 ± 0.1*	2.2 ± 0.2*	WT	WT
16-PLS-33	Resistance	>512	Low	5.1	1.5 ± 0.1*	1.9 ± 0.2*	Q78ter	WT

ZSC-8	Susceptible	8	Low	8.1	1.7 ± 0.1*	1.8 ± 0.2*	Q78ter	WT
582	Susceptible	8	Low	7.2	1.4 ± 0.1*	1.4 ± 0.2*	Q78ter	WT
			
143	Susceptible	8	Intermediate	9.1	1.1 ± 0.1	1.3 ± 0.1	Q78ter	WT
ZSC-12	Susceptible	4	Intermediate	9.5	1.0 ± 0.1	1.0 ± 0.1	Q78ter	WT
1002	Susceptible	4	Intermediate	9.8	1.1 ± 0.2	1.2 ± 0.2	Q78ter	WT
			
916	Susceptible	4	High	10.7	1.1 ± 0.1	1.1 ± 0.1	Q78ter	WT
03-197-1	Susceptible	2	High	11.4	0.9 ± 0.1	0.8 ± 0.1	Q78ter	WT
03-228-1	Susceptible	2	High	10.7	1.0 ± 0.2	1.1 ± 0.1	Q78ter	WT
ZSC-14-1	Susceptible	4	High	11.0	0.9 ± 0.1	1.1 ± 0.1	Q78ter	WT
ZSC-40	Susceptible	4	High	10.5	0.9 ± 0.2	0.9 ± 0.2	WT	WT
419	Susceptible	4	High	12.7	1.0 ± 0.2	0.9 ± 0.1	Q78ter	WT

*MIC, minimum inhibitory concentration; WT, wild-type sequence*.

*^a^MIC break point of nalidixic acid is 32 mg/L*.

*^b^Data from a previous report (Asai et al., 2010)*.

*^c^Data from a previous report (Usui et al., 2009)*.

*^d^Relative expression level compared to the average for six high enrofloxacin-accumulating nalidixic acid-susceptible strains*.

*^e^Q78ter, stop codon mutation, glutamine-78-termination*.

*Indicates mRNA expression is significantly higher than mRNA expression of six high enrofloxacin-accumulating nalidixic acid-susceptible strains (*P* < 0.05).

### Laboratory-selected mutants

Five strains (582, 143, 916, 1002, and 419), susceptible to all of the antimicrobial agents evaluated in this study, were selected as parent strains from which mutants were isolated. Cultures grown on agar containing various agents was employed, including three AcrAB substrates (ampicillin, oxytetracycline, and kanamycin) and one non-substrate (spectinomycin; Bailey et al., 2008; Ricci and Piddock, 2009a,b). For selection of mutants, 0.1 mL of each strain, adjusted to 10^8^–10^9^cfu/mL were exposed to 4 × MIC of the respective substance for each strain in the agar medium, as described previously (Ricci and Piddock, 2009b). A single strain was selected for each combination. In addition, as fluoroquinolone is an AcrAB substrates, enrofloxacin-selected mutants obtained in our previous report were used as control (Usui et al., 2010). MICs of antimicrobials for mutants were determined as described above.

### Quantitative PCR analysis of expression of 16S rRNA, *acrAB*, and multiple global regulators

To evaluate the genes conferring efflux pump activity and their multiple global regulators, quantitative PCR was applied to the detection and quantification of mRNAs (Usui et al., 2010, 2011). The preparation of cDNA was performed as described by Zheng et al. (2009), with slight modifications. In brief, extraction of total RNA from 5 mL of bacterial suspensions was carried out with an ISOGEN kit (Nippon Gene, Tokyo, Japan). cDNA was synthesized from these RNA samples using the PrimeScript RT reagent kit (TaKaRa, Shiga, Japan). Quantitative PCR was performed with SYBR premix EX TaqII (TaKaRa) on a One Step real-time system (Applied Biosystems, Foster City, CA, USA) according to the manufacturer’s instructions. The oligonucleotide primers used for the detection of cDNA are listed in Table 2. The yields of amplicons from *acrA*, *acrB*, *ramA*, *soxS*, *marA*, and *rob* were normalized to those originating from 16S rRNA. Within bacterial cells, 16S rRNA was assumed to be transcribed at a constant rate throughout the growth conditions used in this study.

**Table 2:** **Primers used in this study**.

Primer name	Sequence (5′–3′)	Reference
acrA-F	AAAACGGCAAAGCGAAGGT	Usui et al. (2011)
acrA-R	GTACCGGACTGCGGGAATT	Usui et al. (2011)
acrB-rt1	GGCATTGGGTATGACTGGAC	Zheng et al. (2009)
acrB-rt2	GCATTACGGAGAACGGGATAG	Zheng et al. (2009)
ramA-rt1	TTTCCGCTCAGGTTATCGAC	Zheng et al. (2009)
ramA-rt2	CGGGCAATATCATCAATACG	Zheng et al. (2009)
soxS-rt1	AAATCGGGCTACTCCAAG	Zheng et al. (2009)
soxS-rt2	TACTCGCCTAATGTTTGATG	Zheng et al. (2009)
marA-rt1	ATTCTCTATCTGGCGGAAC	Zheng et al. (2009)
marA-rt2	CGGGTCAATGTTTGCTGTG	Zheng et al. (2009)
robA-rt1	TATTCCGCCAGTGCTTTATG	Zheng et al. (2009)
robA-rt2	CCTGCTCATCGTCTTTCTCC	Zheng et al. (2009)
16S rRNA-F	CCAGCAGCCGCGGTAAT	Usui et al. (2010)
16S rRNA-R	TTTACGCCCAGTAATTCCGATT	Usui et al. (2010)

After normalization to the levels of 16S rRNA, gene expression was compared with the average values for the six high enrofloxacin-accumulating susceptible strains.

### *acrR* DNA sequence analysis

Mutations in the local repressor gene, *acrR*, and the regulator-binding site were detected by direct DNA sequencing, using previously reported primer sets (Zheng et al., 2009). Amplification of the gene and purification of the resulting amplicon were performed as described before (Zheng et al., 2009). Nucleotide sequences were determined using a BigDye Terminator v3.1 Cycle Sequencing Kit (Applied Biosystems) with a 3130 Genetic Analyzer (Applied Biosystems).

### Statistical analysis

Student’s *t*-test was used to compare results between and within experiments. *P*-values < 0.05 were considered significant.

## Results

### Relationship between the enhancement of efflux pumps and the expression of *acrAB*

Expression of *acrA* and *acrB* in the six resistant strains and in the two low enrofloxacin-accumulation nalidixic acid-susceptible strains was significantly higher than that in the other susceptible strains (*P* < 0.05; Table 1). Expression levels of *acrB* correlated significantly with those of *acrA* in this study (correlation coefficient: 0.89).

### Relationship between increased expression of *acrB* and global regulators

**Figure 2:**
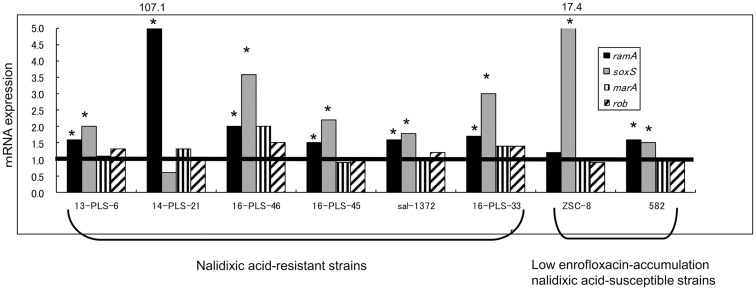
**Expression of multiple regulator genes in field isolates of *S*. Choleraesuis**. The expression of multiple regulator genes in resistant strains and low enrofloxacin-accumulating nalidixic acid-susceptible strains was compared to the average expression of the corresponding genes in high enrofloxacin-accumulating nalidixic acid-susceptible strains (*n* = 6). Horizontal line indicates the average gene expression in the six susceptible strains, which expressions were set to be 1.0. *Indicates that mRNA expression is significantly higher in a strain compared to mRNA expression in the six susceptible strains (*P* < 0.05).

Of the eight strains with a high *acrB* expression level, six strains showed higher levels of both *ramA* and *soxS* expression compared with the average values for the six high accumulating susceptible strains (*P* < 0.05; Figure 2). The remaining strains showed a higher level of *ramA* or *soxS* expression (*P* < 0.05): the 14-PLS-21 strain showed only a higher expression level of *ramA* (107.1-fold ± 42.0-fold), while the ZSC-8 strain showed only a higher expression level of *soxS* (17.4-fold ± 2.7-fold). Neither the expression of *marA* nor that of *rob* changed in any of the strains tested.

### Sequencing of the local repressor gene *acrR* and of the regulator-binding site

A stop codon mutation in AcrR (glutamine-78-to-terminal; Q78ter) was found in 13 strains (Table 1). No significant relationship was found between the Q78ter stop codon mutation in AcrR and the expression of *acrB*. None of the strains tested had a point mutation in the regulator-binding site, which affects the *acrB* expression, previously determined using the *S*. serovar Typhimurium strain BN18 (Olliver et al., 2004).

### Gene expression in laboratory-selected mutants

Expression levels of both *acrB* and *ramA* increased significantly in AcrAB substrate-selected mutants compared to these levels in parental strains (*P* < 0.05; Table 3). Among four AcrAB substrates, a higher level of *ramA* expression was observed in the enrofloxacin- and ampicillin-selected mutants than in the oxytetracycline- and kanamycin-selected mutants (*P* < 0.05). In contrast, the expression of *soxS* decreased significantly in AcrAB substrate-selected mutants compared to that in parental strains (*P* < 0.05; Table 3). MICs of AcrAB substrates were increased in AcrAB substrate-selected mutants as compared with those in the parental strains (Table 3).

**Table 3:** **The MICs of several antimicrobials and gene expressions of *acrB* and multiple regulator genes in laboratory-selected mutants**.

Strain no.	Selecting agent^a^	MIC (mg/L)					mRNA expression^c^				
		Enrofloxacin(2)^b^	Ampicillin (32)^b^	Oxytetracycline (16)^b^	Kanamycin (64)^b^	Spectinomycin(128)^b^	*acrB*	*ramA*	*soxS*	*marA*	*rob*
582		0.0625	1	2	2	32	1.4	1.6	1.5	1.1	1.0
582/E	Enrofloxacin	0.25	4	4	2	32	6.9	39.2	0.8	1.2	0.6
582/A	Ampicillin	0.25	4	8	2	32	7.7	89.5	0.3	1.2	0.8
582/O	Oxytetracycline	0.125	4	4	2	32	4.2	9.5	0.3	1.1	0.9
582/K	Kanamycin	0.125	2	4	4	32	3.1	9.0	0.5	1.3	0.6
582/S	Spectinomycin	0.0625	1	2	2	128	1.7	1.4	1.5	1.2	0.8
143		0.0625	1	2	2	32	1.3	1.2	1.2	1.1	1.0
143E	Enrofloxacin	0.25	4	4	2	32	4.3	45.2	0.6	0.9	0.7
143/A	Ampicillin	0.125	4	4	2	32	2.9	38.8	0.1	0.7	0.5
143/O	Oxytetracycline	0.0625	2	8	2	32	2.2	10.2	1.3	1.2	0.3
143/K	Kanamycin	0.0625	2	4	8	32	2.1	9.3	1.1	1.2	0.4
143/S	Spectinomycin	0.0625	1	2	2	128	1.6	0.9	0.9	0.7	0.7
1002		0.0625	1	2	2	32	1.2	1.0	0.8	1.0	1.0
1002/E	Enrofloxacin	0.25	4	4	2	32	8.0	29.8	0.1	1.2	1.0
1002/A	Ampicillin	0.125	4	2	2	32	2.4	16.1	0.1	0.3	0.5
1002/O	Oxytetracycline	0.125	2	8	2	32	2.2	14.7	0.2	1.2	0.9
1002/K	Kanamycin	0.125	2	4	4	32	2.4	14.2	0.3	1.0	0.9
1002/S	Spectinomycin	0.0625	1	2	2	128	1.1	1.4	0.4	1.1	0.9
916*		0.0625	1	2	2	32	1.1	1.1	0.8	0.9	0.9
916/E	Enrofloxacin	0.5	4	4	2	32	7.4	34.2	0.4	0.8	0.9
916/A	Ampicillin	0.25	4	4	2	32	8.5	58.2	0.1	0.9	0.8
916/O	Oxytetracycline	0.0625	1	4	2	32	2.2	3.5	0.2	1.0	0.9
916/K	Kanamycin	0.0625	1	4	8	32	2.2	6.5	0.1	1.0	0.6
916/S	Spectinomycin	0.0625	1	2	2	128	1.2	0.6	0.2	0.7	0.6
419*		0.0625	4	2	2	32	0.9	0.7	1.0	1.0	1.0
419/E	Enrofloxacin	0.25	16	4	2	32	5.7	24.2	0.2	1.7	0.8
419/A	Ampicillin	0.0625	8	2	2	32	1.6	32.5	0.3	0.7	0.9
419/O	Oxytetracycline	0.125	8	8	2	32	3.0	21.8	0.8	3.4	1.6
419/K	Kanamycin	0.0625	8	4	8	32	1.5	18.5	0.3	1.1	0.9
419/S	Spectinomycin	0.0625	4	2	2	128	0.9	1.1	0.4	2.1	1.2
03-197-1*		0.0625	>128	256	2	32	0.8	1.1	1.3	1.2	1.2
03-228-1*		0.0625	1	256	8	32	1.1	1.1	1.0	0.9	0.9
ZSC-14-1*		0.0625	1	256	2	32	1.1	1.0	1.0	1.0	0.8
ZSC-40*		0.0625	>128	256	2	32	0.9	0.8	0.8	1.1	0.9

*MIC, minimum inhibitory concentration*.

*^a^Mutants were selected by exposing to fourth the MIC of the antimicrobials for each susceptible strain*.

*^b^MIC data of antimicrobials except for spectinomycin in parental strains from a previous report (Asai et al., 2010). Parenthesis indicates break points*.

*^c^Relative expression level of acrB and multiple regulator genes compared to average of six high accumulation susceptible strains represented as asterisks*.

Expression of *acrB* and multiple regulator genes remained stable in spectinomycin-selected mutants. In spectinomycin-selected mutants, MICs of AcrAB substrates were of the same level as compared with those in the parental strains (Table 3).

## Discussion

The current study showed that the expression of *ramA* and/or *soxS* was associated with *acrAB* overexpression in field isolates of *S*. Choleraesuis. In this study, expression of *ramA*, but not *soxS*, *marA*, or *rob*, was enhanced in laboratory-derived *S*. Choleraesuis mutants selected using antimicrobials that are AcrAB substrates. Several previous studies have reported that *ramA*, but not *soxS*, is involved in activation of the AcrAB-TolC system in *S*. Typhimurium (Ricci et al., 2006; Bailey et al., 2008; Zheng et al., 2009). Interestingly, in this study, *soxS* expression was downregulated in AcrAB substrate-selected mutants of *S*. Choleraesuis. In addition, enrofloxacin- and ampicillin-selected mutants derived from the ZSC-8 strain with a high level of *soxS* expression, showed increased expression of *ramA*, but not of *soxS* (data not shown). Several studies have suggested that increased expression of *ramA* downregulates *soxS* expression (Nikaido et al., 2008; O’Regan et al., 2009). These results suggest that the *acrA* and *acrB* expression in antimicrobial-selected mutants of *S*. Choleraesuis were dependent on *ramA* expression.

The present study showed that increased levels of both *ramA* and *soxS* expression were demonstrated by some field isolates of *S*. Choleraesuis upon activation of the AcrAB-TolC system. There was a marked difference in *soxS* expression between the field isolates and the laboratory-derived strains. As all of the strains used in this study were isolated from diseased pigs, these isolates would have been exposed to antimicrobials and disinfectants used in disease treatment and hygiene management. This suggests that the resistance in some field isolates was mediated through a different mechanism, and therefore could have arisen from a different selective pressure.

Activation of the AcrAB efflux pump is responsible for the emergence of fluoroquinolone-resistant *Salmonella* strains (Ricci et al., 2006; Usui et al., 2009). In Japan, the description of veterinary fluoroquinolone drugs includes an explanation that the drug is considered as a second-line drug. In the AcrAB substrate-selected mutants, the level of *acrB* expression generally increased, depending on *ramA* expression. It is possible that the use of an AcrAB substrate as a first-line drug crucially selects strains of *S*. Choleraesuis with activated AcrAB efflux systems. Among AcrAB substrates used in this study, the degree of *acrB* expression may be different due to exposure to the various antimicrobial agents. Therefore, the antimicrobial class used as first-line drugs may be associated with the frequency of fluoroquinolone resistance in bacterial strains. Further studies are needed in order to clarify the effects of each antimicrobial on *acrB* expression in these bacteria.

In conclusion, the AcrAB-TolC efflux system in field isolates of *S*. Choleraesuis is likely regulated by external factors in the environment, such as antimicrobials and drug residues *via* *ramA* and *soxS*. In particular, exposure of AcrAB substrates enhances activation of AcrAB *via* RamA in *S*. Choleraesuis.

## Conflict of Interest Statement

The authors declare that the research was conducted in the absence of any commercial or financial relationships that could be construed as a potential conflict of interest.
